# A Mechanistic Understanding of Axon Degeneration in Chemotherapy-Induced Peripheral Neuropathy

**DOI:** 10.3389/fnins.2017.00481

**Published:** 2017-08-31

**Authors:** Yusuke Fukuda, Yihang Li, Rosalind A. Segal

**Affiliations:** ^1^Department of Neurobiology, Harvard Medical School Boston, MA, United States; ^2^Department of Cancer Biology, Dana-Farber Cancer Institute Boston, MA, United States

**Keywords:** axon, chemotherapy, CIPN, degeneration, DRG, neuropathy, sensory neuron, Wallerian

## Abstract

Chemotherapeutic agents cause many short and long term toxic side effects to peripheral nervous system (PNS) that drastically alter quality of life. Chemotherapy-induced peripheral neuropathy (CIPN) is a common and enduring disorder caused by several anti-neoplastic agents. CIPN typically presents with neuropathic pain, numbness of distal extremities, and/or oversensitivity to thermal or mechanical stimuli. This adverse side effect often requires a reduction in chemotherapy dosage or even discontinuation of treatment. Currently there are no effective treatment options for CIPN. While the underlying mechanisms for CIPN are not understood, current data identify a “dying back” axon degeneration of distal nerve endings as the major pathology in this disorder. Therefore, mechanistic understanding of axon degeneration will provide insights into the pathway and molecular players responsible for CIPN. Here, we review recent findings that expand our understanding of the pathogenesis of CIPN and discuss pathways that may be shared with the axonal degeneration that occurs during developmental axon pruning and during injury-induced Wallerian degeneration. These mechanistic insights provide new avenues for development of therapies to prevent or treat CIPN.

## Introduction

Cancer therapies result in multiple toxic side effects that limit the doses used and cause long-lasting damage to patients. Chemotherapy-induced peripheral neuropathy (CIPN) is a severe and long lasting side effect caused by diverse anticancer agents that damage sensory and/or motor nerves. Symptoms of CIPN include numbness, pain, burning, tingling, heat/cold hyperalgesia, and mechanical allodynia, as well as reduced motor function. CIPN commonly presents with a “Glove-and-Stocking” distribution with the most distal portions of the limb exhibiting the greatest deficits (Brewer et al., 2016). CIPN occurs in 30–70% of patients treated with specific categories of anticancer agents (Seretny et al., 2014). Symptoms usually begin after multiple doses of the chemotherapeutic agents, and progress as treatment continues. After the treatments cease, they can resolve in a short time period, or persist as a long-lasting sequela of cancer therapy.

Clinical assessment of CIPN is usually based on patient-derived questionnaires and physician-based grading scales such as the common toxicity criteria (CTC) scale. These evaluation methods suffer from subjectivity and inconsistency (Cavaletti et al., 2010; Brewer et al., 2016). More objective evaluation methods have also been developed, such as quantitative sensory testing (QST), which measures the detection threshold for both mechanical and thermal sensory inputs. Nerve conduction studies (NCS), which assess both sensory and motor nerve action potential can also be used in patient assessment (Brewer et al., 2016), as decreased amplitude of sensory action potential are commonly observed in CIPN patients (Chaudhry et al., 1994; Park et al., 2013). Skin biopsy and quantitative assessment of intraepidermal nerve fiber (IENF) density provides a sensitive, objective and quantitative measurement of the small nerve fiber neuropathy commonly seen in CIPN (Periquet et al., 1999; Kroigard et al., 2014). However, these more objective measurements are not easily deployed in clinical assessments.

## Chemotherapeutic drugs that cause CIPN

Multiple anticancer drugs cause peripheral neuropathies; the most common are the taxanes, vinca alkaloids, platinum-based drugs, and protease inhibitors (Brewer et al., 2016; Table 1). Taxane agents (e.g., paclitaxel and docetaxel) exert their antimitotic effect by binding to polymerized tubulin within microtubules, and thereby preventing microtubule depolymerization (Jordan and Wilson, 2004). Vinca alkaloids (e.g., vincristine and vinblastine), another class of agents targeting microtubules, promote microtubule depolymerization and thereby disrupt mitotic spindles and cause cell cycle arrest (Jordan and Wilson, 2004; Kavallaris, 2010). As highly polarized dorsal root ganglion (DRG) sensory neurons require proper microtubule dynamics for axonal transport of mRNAs, proteins, mitochondria and other organelles, it is perhaps not surprising that these microtubule binding agents can cause degeneration of peripheral nerve fibers and symptoms of CIPN (Authier et al., 2000; Ja'afer et al., 2006; Gornstein and Schwarz, 2014; Geisler et al., 2016). As these two drugs with opposite effects on microtubule stability both can cause CIPN, it appears likely that diverse perturbations in microtubule dynamics may play a critical role instigating CIPN.

**Table 1:** Chemotherapeutic drugs implicated in CIPN.

**Type of drug**	**Example**	**Mechanisms of drug action in treating cancer**	**Type of neuron affected**
Platinum agents	Cisplatin Oxaliplatin	Bind to DNA, cell cycle arrest and apoptosis	Sensory
Taxane	Paclitaxel	Inhibit microtubule depolymerization, mitotic arrest	Sensory
Vinca alkaloid	Vincristine	Inhibit microtubule polymerization, mitotic arrest	Sensory, as well as motor and autonomic
Proteasome inhibitor	Bortezomib	Inhibit proteasome degradation, cycle arrest; enhance microtubule polymerization	Sensory

The platinum-based chemotherapeutics (e.g., cisplatin and oxaliplatin) exert their antineoplastic activity by forming interstrand DNA adduct (Suchankova et al., 2012), leading to cell cycle arrest (Johnstone et al., 2014). Patients treated with platinum agents exhibit predominantly sensory neuropathy (Addington and Freimer, 2016). It is not known whether this sensory neuropathy is due to damage of nuclear or mitochondrial DNA in DRG neurons or other cells, or reflects other actions of platinum-based compounds.

Proteasome inhibitors represent a novel class of anticancer drugs that result in protein accumulation and apoptosis (Adams, 2004). Bortezomib, an inhibitor of the 20S subunit of the proteasome, was the first proteasome inhibitor approved for clinical treatment of multiple myeloma (Curran and McKeage, 2009). Bortezomib induces a peripheral sensory neuropathy. As bortezomib exerts a microtubule stabilizing activity similar to paclitaxel in addition to proteasome inhibition (Poruchynsky et al., 2008), it is not clear whether the neuropathic effect reflects proteasome inhibition or microtubule changes.

While other agents have been reported to cause neuropathy in some patients, the above chemotherapeutic agents represent the major drug categories currently responsible for CIPN. These agents have different mechanisms of action, and so it is not known whether they cause CIPN by a common pathway. Given the poor understanding of the disorder, it is not surprising that there are currently no effective treatments of CIPN, and that multiple clinical trials have had disappointing results. Among recent trials, duloxetine, an antidepressant therapy that inhibits serotonin and norepinephrine reuptake, was shown to have a small, but significant beneficial effect on CIPN (Smith et al., 2013). However, the other 14 out of 15 therapies tested failed to show beneficial effects on CIPN patients (Majithia et al., 2016). Thus, there is a clear need for greater understanding of CIPN to enable development and testing of new therapies. This review summarizes the up-to-date understanding of CIPN, discusses the known mechanisms of general axon degeneration and how this understanding provides insight into for future research into the pathogenesis of CIPN.

## Pre-clinical models for studying CIPN

### Animal models

To understand the biology of CIPN, a variety of preclinical models have been developed. Chemotherapy drugs are typically given to rodents (rats or mice) through intraperitoneal or intravenous injection, followed by behavioral, electrophysiological, or morphological analysis. These models enable a rigorous assessment of the neuropathological features consistently observed following chemotherapeutic treatment. A recent review (Hoke and Ray, 2014) provides a thorough overview of the current animal models used for CIPN study.

A “dying back” axon degeneration is a prototypical pathological feature of CIPN in patients, and this can be assessed both in patients and in rodent models by skin biopsy and measurement of IENF density (Authier et al., 2000). Both the number of innervating nerve fibers and the neuronal subtypes perturbed by chemotherapeutic agents can be readily and quantitatively assessed in animal models. Additional studies of sensory and motor nerve conduction including sensory and compound muscle action potentials and latency of evoked response can be carried out to assess CIPN in animal models. Several groups have reported decreased sciatic nerve fiber diameter in rats treated with various dosages of paclitaxel (Authier et al., 2000; Persohn et al., 2005; Arrieta et al., 2011), as well as decreased peripheral nerve conduction (Persohn et al., 2005); however motor function is not altered (Authier et al., 2000). Peripheral nerve fiber degeneration has also been reported in rats/mice treated with vincristine (Ja'afer et al., 2006; Geisler et al., 2016), bortezomib (Cavaletti et al., 2007; Carozzi et al., 2010; Meregalli et al., 2010) and cisplatin (Carozzi et al., 2010; Arrieta et al., 2011). Although sensory neurons represent the most common target of CIPN, damage to motor and autonomic neurons have also been reported (Mora et al., 2016).

Behavioral studies in rodent models of CIPN assess mechanical allodynia, mechanical hyperalgesia, and thermal hypo- and hyperalgesia following exposure to chemotherapeutic drugs. Mechanical allodynia, the over sensitization to touch, is assessed by applying a series of monofilaments, the Von Frey filaments, to the hind paw of the animal and the threshold of pressure applied that causes the animals to withdraw their paw is measured. To measure changes in responses to temperature, a hot or cold stimulus is applied by tail immersion or radiant heat, and the response as assessed by tail flick or paw withdrawal is quantified. It is not yet known which of these behaviors best reflects the clinical condition. Thermal hyperalgesia is commonly observed in rodent models of CIPN (Authier et al., 2000; Cata et al., 2008; Zheng et al., 2012), while mechanical hyperalgesia (Authier et al., 2000) and thermal allodynia (Arrieta et al., 2011) are also reported. Unfortunately, the behaviors observed are inconsistent among different research groups, which is likely due to the fact that current rodent models are not standardized for animal strain, age, sex, or drug dosing schedule (Hoke and Ray, 2014). The diverse methods of initiating and assessing CIPN in rodent models make it difficult to compare data across laboratories.

Non-mammalian models have also been reported, but are less commonly studied. Lisse and colleagues reported on a Zebrafish model, in which DRG axon degeneration and impaired twitching response were observed after a 4-day treatment with paclitaxel (Lisse et al., 2016). Several groups have also established *Drosophila* models to study the underlying mechanisms of CIPN. To establish these models, adult flies or larvae are fed with food that contains chemotherapeutic drugs. *Drosophila* larvae fed paclitaxel exhibited axonal swellings and axon loss in sensory neurons without alterations at the neuromuscular junction (Bhattacharya et al., 2012). In this *Drosophila* model, axon degeneration occurs without loss of the neuronal cell bodies. Interestingly, overexpression of NMNAT, a protective protein during axon injury, prevented this paclitaxel-induced axon degeneration. Cisplatin fed adult *Drosophila* exhibited enhanced neuronal apoptosis in the brain and displayed defective climbing behavior (Podratz et al., 2011b, 2013). These results are consistent with findings from rodent models and from people, suggesting that these non-mammalian systems can provide valuable models to study CIPN.

### Tissue culture models

In addition to animal models, tissue culture preparations of rat and mouse DRG sensory neurons are used to study CIPN at a mechanistic level (Malgrange et al., 1994; Yang et al., 2009; Guo et al., 2017). While most studies have relied on embryonic tissue, a recent study by Gornstein et al. developed a DRG culture from adult mice to study paclitaxel-induced neuropathy (Gornstein and Schwarz, 2017). Recent methods for turning human adult cells into induced pluripotent stem cells (iPSCs) and reprogramming these cells to generate peripheral sensory neurons have enabled studies of CIPN on cultured human cells (Chambers et al., 2012; Wainger et al., 2015). A recent study analyzed the effects on different chemotherapy agents on neurite outgrowth using commercially available iPSC-derived neurons coupled with high content imaging analysis. Both paclitaxel and vincristine-treated neurons showed decreased neurite outgrowth without increased cell death, while cisplatin treatment induced cell death (Wheeler et al., 2015). Moreover, knocking down *TUBB2A*, a gene encoding a tubulin isoform that has previously been identified as the locus for a single nucleotide polymorphism associated with enhanced risk of CIPN (Leandro-Garcia et al., 2012), increased the sensitivity of iPSC-derived neurons to paclitaxel treatment (Wheeler et al., 2015). This result supports the hypothesis that disruption of microtubule dynamics may be one mechanism contributing to CIPN. These findings also suggest that human iPSC-induced neurons can provide a reliable and powerful *in vitro* human model for mechanistic and therapeutic studies of CIPN.

## Pathology of CIPN

**Figure 1:**
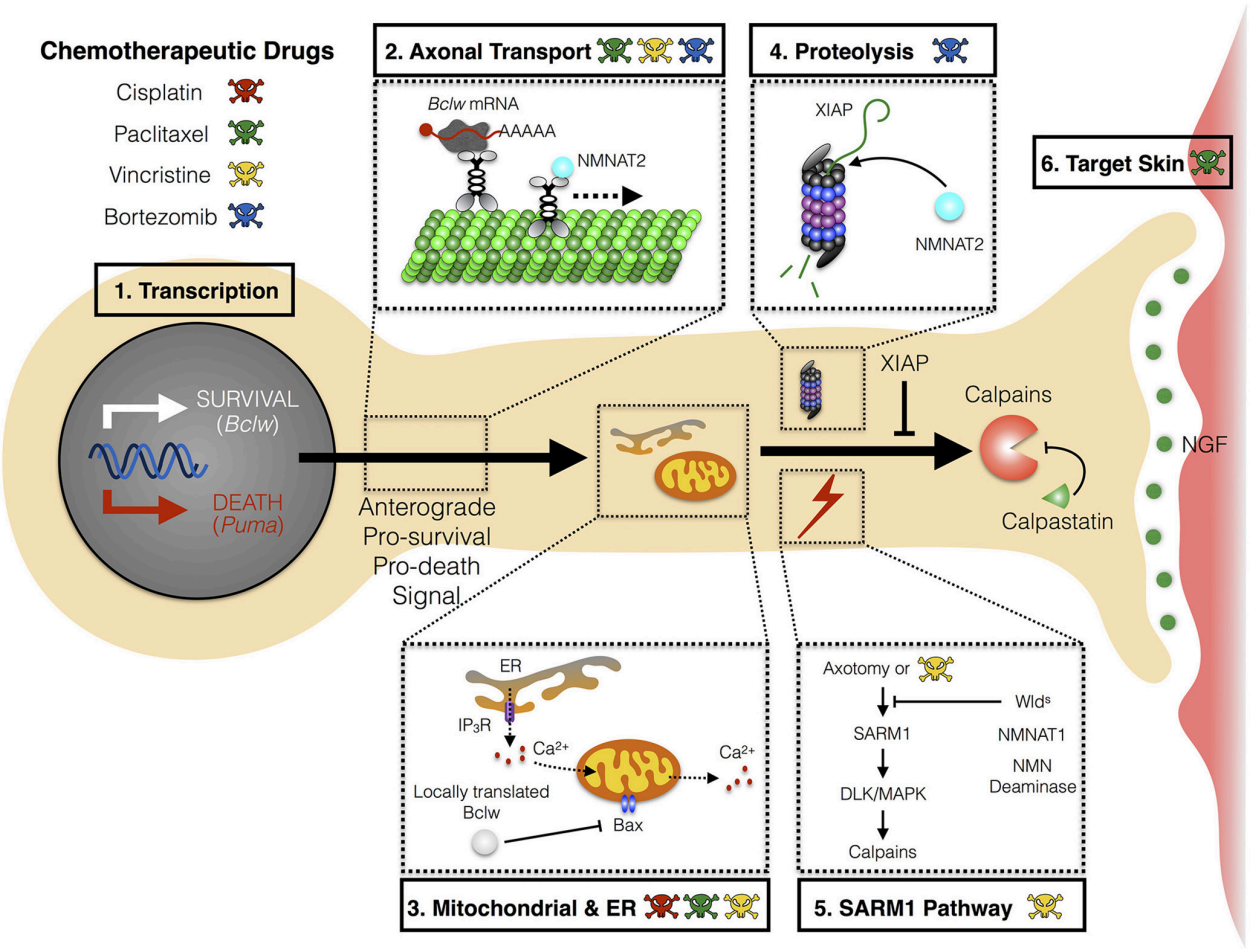
Mechanism of axon degeneration and sites of action of chemotherapeutic drugs. Axon degeneration is determined by factors that can either enhance death and/or inhibit survival pathways. Key mechanisms that underlie this pathway are transcriptional programs, microtubule-dependent transport, local translation to modulate mitochondrial function, and proteolysis. Chemotherapeutic agents (illustrated by colored skull and cross bones) that are currently known to modulate these key mechanisms are depicted (see text for detail). **(1)** During developmental axon pruning, target-derived neurotrophins instruct transcription of key pro-survival genes (*Bclw*). Under neurotrophic deprivation, the function of pro-survival genes is overcome by induction of pro-death genes (*Puma*). **(2)** Key transcripts of neurotrophin-dependent retrograde response genes (*Bclw*) are transported in an anterograde manner by kinesin motor proteins toward distal axons. Cell soma-derived factors (NMNAT2) are also transported to axons to replenish axonal pools of proteins with fast turnover rate. **(3)** Local translation of Bclw and LaminB2 (not shown) modulates mitochondrial function to inhibit the caspase cascade. Mitochondrial and ER integrity is also critical for maintaining Ca^2+^ homeostasis to prevent activation of Ca^2+^-dependent calpains. **(4)** Neurotrophic deprivation leads to degradation of XIAP, a key inhibitor of the caspase cascade. NMNAT2, a survival factor with a short half-life, is possibly degraded through the proteasome in axons. **(5)** Vincristine and axotomy activates SARM1 and DLK/MAPK signaling, leading to activation of calpains, the ultimate convergence point for executing axon degeneration. Expression of Wld^s^, axonal NMNAT1, or NMN deaminase provides protection against vincristine or injury-induced degeneration. **(6)** Non neuron-autonomous effects from other cell types can potentially sensitize axons to degeneration during CIPN. Paclitaxel-induced neurotoxicity can mediate inflammatory response and epithelial damage to perturb skin homeostasis. Bclw, Bcl-2-like protein 2; Puma, p53 upregulated modulator of apoptosis; NMNAT, nicotinamide mononucleotide adenylyltransferase; ER, endoplasmic reticulum; IP_3_R, inositol 1,4,5-triphosphate receptor; BAX, Bcl-2-like protein 4; XIAP, X-linked inhibitor of apoptosis protein; SARM1, sterile α-motif-containing and armadillo-motif-containing protein; DLK, dual leucine zipper kinase; MAPK, mitogen-activated protein kinase; Wld^s^, Wallerian degeneration slow; NMN, nicotinamide mononucleotide; CIPN, chemotherapy-induced peripheral neuropathy.

The mechanism(s) whereby chemotherapies cause CIPN are not yet understood, nor is it clear whether distinct agents converge on a shared pathway to induce symptoms. While effects of chemotherapeutic agents on neurons, glial cells, and skin cells have each been suggested to initiate the neurological symptoms, studies in patients and in animal models implicate axonal degeneration as a common process in CIPN pathology. Specifically, chemotherapeutic agents either directly or indirectly trigger a “dying back” axon degeneration that proceeds in a distal-to-proximal manner. Potential mechanisms for initiating axon degeneration include defects in axon transport, altered mitochondrial function, or altered Ca^+2^ homeostasis (Figure 1).

### CIPN and axon transport

Paclitaxel, vincristine, and bortezomib all affect microtubule dynamics by inhibiting the processes of tubulin depolymerization and polymerization (Figure 1). Paclitaxel causes retraction bulbs at the tips of sensory nerve axons, indicating axon degeneration (Gornstein and Schwarz, 2017). Epothilone B, a chemotherapy drug currently in clinical trials, is a structurally distinct compound that binds to the same site on tubulin as paclitaxel. Interestingly, DRG cultures treated with epothilone B showed a similar axon tip retraction as cultures treated with paclitaxel (Gornstein and Schwarz, 2017). This further indicates that altered microtubule dynamics contributes to taxane-induced axonopathy. As both anterograde and retrograde axonal transport rely on microtubule integrity and dynamics, CIPN drugs targeting microtubule are likely to result in altered axonal transport of essential cellular components. LaPointe and colleagues used an *in vitro* vesicle motility assay and discovered that vincristine and paclitaxel inhibit anterograde axonal transport in axoplasm isolated from squid giant axons, and that vincristine inhibits retrograde transport as well (LaPointe et al., 2013). Additional studies in simple systems affirm that paclitaxel can reduce axonal transport (Theiss and Meller, 2000; Shemesh and Spira, 2010). In support of the possibility that impaired transport causes CIPN, a recent study demonstrated reductions in the levels of several axonal mRNAs in the distal nerves of mice treated with paclitaxel (Bobylev et al., 2015). In contrast, time lapse studies detected little change in mitochondria and late endosome/lysosome transport along microtubules following paclitaxel treatment (Gornstein and Schwarz, 2017). Thus, it is possible that paclitaxel causes defective mRNA transport, rather than a general disruption of microtubule-based motility.

### Mitotoxicity in CIPN

Mitochondrial dysfunction is commonly observed in CIPN (Figure 1). Abnormal mitochondrial morphology including swelling, vacuolation, enlargement, and loss of cristae structure have been observed in peripheral nerve axons (Flatters and Bennett, 2006; Xiao et al., 2012; Zheng et al., 2012; Bobylev et al., 2017), but not in the surrounding Schwann cells (Xiao et al., 2012) in several CIPN animal models. Potential causes of altered mitochondria include paclitaxel-induced depletion of mRNAs encoding mitochondrial fission/fusion machinery in distal axons (Bobylev et al., 2015). Mitochondria dynamics may be altered in cisplatin-treated animals due to decreased level of mitochondrial fusion protein MFN2 in distal axon segments (Bobylev et al., 2017). Moreover, increased mitochondrial fragmentation, as well as decreased mitochondrial fission/fusion dynamics and motility, occur rapidly in vincristine-treated DRG cultures and precede axon degeneration (Berbusse et al., 2016). Platinum agents can also affect mitochondrial function through damaging mitochondrial DNA (mtDNA), and thereby impeding mtDNA replication and transcription (Podratz et al., 2011a). Thus, mitochondrial dysfunction may represent a common mechanism for axon degeneration in CIPN (Figure 1).

### Ca^2+^ homeostasis and cation channels

Ca^2+^ homeostasis is critical for neuronal and axonal health. Mitochondria and the endoplasmic reticulum (ER) both function as intracellular stores of Ca^2+^, and paclitaxel treatment alters Ca^2+^ homeostasis, potentially by inducing Ca^2+^ release from the mitochondria (Kidd et al., 2002; Figure 1). An alternative mechanism for alterations in intracellular Ca^2+^ dynamics was presented by Boehmerle and colleagues, who reported that paclitaxel-induced Ca^2+^ oscillation reflects altered function of inositol 1,4,5-trisphosphate receptors (IP_3_R) in the ER (Boehmerle et al., 2006; Figure 1). Indeed, chronic paclitaxel exposure leads to impaired phosphoinositide-mediated Ca^2+^ signaling in both neuroblastoma cell and DRG cultures (Boehmerle et al., 2007). Increased intracellular Ca^2+^ can activate the potent protease calpain, which directly triggers axon degeneration (Wang et al., 2012). Moreover, Ca^2+^ reducing drugs alleviate mechanical allodynia and hyperalgesia in paclitaxel and vincristine rat models (Siau and Bennett, 2006).

Chemotherapeutic agents may alter cation channels more generally. Recent studies showed that cisplatin, paclitaxel or bortezomib treatment result in increased expression of the non-selective cation channels, TRPV1 and TRPA1, in cultured DRG neurons (Ta et al., 2010; Hara et al., 2013; Quartu et al., 2014). As TRP channels are critical for pain signaling, alterations in these cation channels may be an important component of CIPN. Therefore, approaches that target Ca^2+^ homeostasis and/or cation channels provide potential future therapies.

### Central sensitization and CNS glial activation

While abundant data indicate that peripheral nerves and epidermal innervation are affected in CIPN, changes in the central nervous system (CNS) may also contribute to the disorder. Central sensitization may occur as a direct or indirect consequence of chemotherapeutic agents. Low concentrations of chemotherapeutic drugs, oxaliplatin, and paclitaxel, can be detected in the CNS after systemic dosing that produce hyperalgesia in rats (Huang et al., 2016). Paclitaxel can directly sensitize spinal neurons to TRPV1-mediated capsaicin response (Li et al., 2015), while oxaliplatin can increase expression and release of chemokine C-X3-C motif ligand 1 from spinal cord neurons (Huang et al., 2016). Thus, chemotherapeutic agents can possibly act directly on spinal neurons to mediate central sensitization.

The release of chemokines and cytokines from spinal neurons may further potentiate neuropathic pain by activating microglia and astrocytes. Paclitaxel can induce activation of astrocytes, and in some cases microglia, in the dorsal horn of the spinal cord (Zhang et al., 2012; Ruiz-Medina et al., 2013). Consistent with these effects, drugs that affect astrocytes and microglia, including cannabinoid agonists and minocycline, have been reported to alleviate mechanical allodynia in rats (Boyette-Davis et al., 2011; Burgos et al., 2012). Interestingly, some chemotherapeutics can also activate astrocytes and microglia in regions of the brain associated with chronic pain and nociceptive processing, as paclitaxel increases expression of GFAP in anterior cingulate cortex concurrent with behavioral evidence of thermal hyperalgesia (Masocha, 2015). Collectively, these evidences demonstrate a potential contribution of CNS sensitization and glial activation to the neuropathic pain that occurs in CIPN.

## Mechanistic insights into CIPN and axon degeneration

The recent failures of multiple clinical trials for CIPN (Majithia et al., 2016) underscore the need to explore and address underlying molecular mechanisms more broadly. Since all the relevant chemotherapeutic agents, either directly or indirectly, trigger a “dying back” axon degeneration, a greater understanding of the biology of axon degeneration process *per-se* can identify major players and signaling pathways that can be targeted. Axon degeneration can be provoked by multiple stimuli in addition to chemotherapeutic agents. Axon pruning during development and axotomy also entail axon degeneration. Here we will discuss what is currently known about the cellular pathways for axon degeneration in these contexts, to provide insights for addressing the molecular mechanisms of axon degeneration during CIPN.

### Developmental axon pruning

Axon degeneration constitutes a necessary and healthy end result of pruning that allows plasticity of neuronal circuitry during development. The process of axon pruning has been studied in diverse species including mice, *Drosophila, C. elegans*, and zebrafish (Kage et al., 2005; Hayashi et al., 2009; Poulain and Chien, 2013; Yu and Schuldiner, 2014; Riccomagno and Kolodkin, 2015). Early in development, excess innervation can be observed in both the CNS and PNS. Subsequently, refinement of neuronal connectivity through axon pruning enables the establishment of the robust mature circuitry. Multiple signaling processes critical for axon pruning and refinement have been identified in studies of both central and peripheral neurons.

Repulsive axon guidance cues such as the semaphorins bind to pruning receptors and initiate local axon degeneration in the CNS. For example, in the hippocampus, stereotyped pruning of infrapyramidal bundle (IPB) is initiated by semaphorin3F expressed in interneurons in the stratum oriens, and is achieved by signaling complexes containing neuropilin-2 and plexin-A3 (Bagri et al., 2003). A similar role for semaphorin3F in axon pruning has been observed in corticospinal tract from layer 5 of the visual cortex (Low et al., 2008). In *Drosophila*, mushroom body (MB) γ neurons provide another system where stereotyped axon pruning occurs during development. Pruning of dorsal and medial axonal branches during metamorphosis requires signaling through a TGF-β receptor complex in MB γ neurons, and is initiated by myoglianin secreted from nearby glial cells (Awasaki et al., 2011). Although TGF-β signaling is essential for axon fragmentation, it is not sufficient. Thus, glial cells initiate the process of axon fragmentation in these cells but other factors are required to fully execute axon pruning. Similarly, chemotherapeutic agents acting directly on nearby glial cells may sensitize neurons subsequently undergo axon degeneration. Although paclitaxel has been shown to induce inflammatory responses and promote epithelial damage prior to the induction of axon degeneration (Lisse et al., 2016; Zhang et al., 2016), this model of sensitization has not yet been explored with other chemotherapeutic drugs.

Much of our understanding of developmental axon pruning is derived from studies of neurotrophins, nerve growth factor (NGF), brain-derived neurotrophic factor (BDNF) and neurotrophins 3 and 4 (NT3 and NT4), which are released in a limited amount by target tissues and promote axon and cell body survival (Harrington and Ginty, 2013). The role of neurotrophins in regulating axonal survival and degeneration is most widely studied in the PNS, particularly sympathetic and sensory neurons. Target-derived neurotrophins bind and activate tropomyosin receptor kinase (Trk) receptors in the innervating axon terminals. Endocytosis of the activated receptors results in signaling endosomes that are retrogradely trafficked along the axons. The signaling endosomes initiate instructive programs that promote both axonal and cell survival (Tasdemir-Yilmaz and Segal, 2016). Consequently, neurons that fail to receive neurotrophins lose their innervation, and may undergo apoptosis. This selection process of both axonal and cell degeneration is further enhanced by active signaling to eliminate axon processes. In superior cervical ganglion (SCG) sympathetic neurons, active signaling through the p75 neurotrophin receptor (p75NTR) can promote axon degeneration or even apoptosis (Singh et al., 2008). These examples provide evidence of overlap between cellular and axonal survival and death processes, and suggest that cumulative responses to pro-survival and pro-death signals may also play a role in the axon degeneration observed in CIPN.

### Regulation of axonal survival during development: coordination of transcription, transport, translation, and proteolysis

Regulation occurs at multiple levels to determine whether axons will survive or degenerate, including nuclear transcription, axonal translation and protein activity (Tasdemir-Yilmaz and Segal, 2016). *In vitro* culture systems using compartmented culture platforms have proven to be valuable tools that recapitulate the spatially and fluidically isolated cell body and distal axon compartment observed *in vivo*, enabling mechanistic understanding of the multiple regulatory steps involved in determining and implementing axonal survival or degeneration.

In these compartmented systems, stimulation of axon terminals with target-derived neurotrophins promotes axon outgrowth and prevents both axon degeneration and cell body apoptosis. In contrast, neurotrophin stimulation of cell bodies prevents apoptosis, but does not prevent axon degeneration. Therefore, some of the retrograde response genes that are selectively transcribed in response to neurotrophin stimulation of axons and not by stimulation of cell bodies are likely to encode components needed for axonal survival. These transcriptional changes can be closely linked to events in the axon, as many newly transcribed mRNAs are transported to the distal axons, where they are translated into protein. For example, the Bcl2 family member, *Bclw* (aka *Bcl2l2*) is a retrograde response gene, and newly transcribed *Bclw* mRNA is transported to the axons, where it is locally translated and the resultant protein promotes axonal survival (Pazyra-Murphy et al., 2009; Courchesne et al., 2011; Cosker et al., 2013; Figure 1).

Recently, the RNA binding protein SFPQ was shown to bind multiple axonal transcripts including *Bclw* and *LaminB2* mRNAs (Cosker et al., 2016). While LaminB2 is predominantly a nuclear membrane protein, locally translated LaminB2 associates with axonal mitochondria and adjusts mitochondrial function (Yoon et al., 2012). SFPQ is needed for transport and axonal localization of *Bclw, LaminB2*, and others, and so functions to promote axonal survival and prevent axon degeneration (Cosker et al., 2016; Thomas-Jinu et al., 2017). Together these studies demonstrate that regulated transcription, axonal transport, and local translation work in concert to determine and implement axon survival pathways. Currently, there is no evidence for chemotherapeutic agents affecting transcription of pro-survival genes or translation of axonal mRNAs. However, as this pathway is critical in axon survival, such studies represent an important direction for future research.

In addition to the neurotrophin-mediated axon survival pathways above, key transcriptional programs have recently been uncovered that actively promote axon degeneration during trophic deprivation. Simon et al. demonstrated that loss of neurotrophic support induces transcription of *Puma*, which encodes a pro-apoptotic BH3-only family protein Puma, through the DLK/MAPK signaling pathway (Simon et al., 2016; Figure 1). Maor-Nof et al. provided further mechanistic insight into this transcriptional program, demonstrating that the phosphatase Dusp16 functions to put a brake on the degenerative response by inhibiting *Puma* transcription (Maor-Nof et al., 2016). At present it is not clear whether the Puma protein subsequently functions in the cell bodies or in axons to trigger axon degeneration. In either case, it will be important to assess whether this pro-degenerative component contributes to the biology of CIPN.

A final step in executing axon degeneration involves proteolytic cleavage of axonal proteins by caspases and calpains. Initiator caspase-9 and the effector caspases-3 and -6 have all been implicated in axon degenerative cascades (Nikolaev et al., 2009; Schoenmann et al., 2010; Simon et al., 2012; Cusack et al., 2013; Unsain et al., 2013). Caspase-mediated degeneration is usually held in check by inhibitor of apoptosis protein (IAP) (Figure 1) (Potts et al., 2003; Cusack et al., 2013; Unsain et al., 2013). Calpains are Ca^2+^-activated proteases that execute axonal degeneration by proteolysis of multiple cellular proteins. Calpastatin inhibits calpain activation during developmental pruning and so limits the extent of axon degeneration (Yang et al., 2013). Similarly, calpain-mediated proteolysis has been implicated as a convergent pathway that contributes to axon degeneration in CIPN (Figure 1). Mice treated with paclitaxel that are administered calpain inhibitors show reduced signs of axonal degeneration in sensory neurons and improved clinical measures of neuropathy (Wang et al., 2004) One of the targets of calpain during paclitaxel-induced peripheral neuropathy is neuronal calcium sensor-1 (NCS-1). NCS-1 binds to IP_3_R to enhance intracellular calcium signaling. Paclitaxel disrupts IP_3_R-mediated intracellular calcium signaling via NCS-1 degradation (Boehmerle et al., 2007). In the future, it will be important to identify other molecular downstream targets of calpain relevant to peripheral neuropathy. Nonetheless, these studies demonstrate calpain as a promising therapeutic target for CIPN.

### Wallerian degeneration

Axon degeneration due to traumatic injury represents another useful model for mechanistic studies of axon degeneration or survival. Following axotomy, an initial latent phase is followed by fragmentation of the cytoskeleton, destruction of organelles, and ultimately disintegration of axons distal to the severed site (Gerdts et al., 2016). This process of axon degeneration is referred to as Wallerian degeneration (WD). Much of the mechanistic understanding of WD derives from a spontaneous genetic mutation, Wallerian degeneration slow (*Wld*^s^), which delays the process of WD. This mutation encodes a chimeric fusion protein of the N-terminal fragment of E4 ubiquitin ligase Ube4b and the enzyme nicotinamide mononucleotide adenylyltransferase 1 (NMNAT1; Conforti et al., 2000). The axonal protective effect of the mutant Wld^s^ depends on the NMNAT1 portion of the fusion protein, which synthesizes nicotinamide adenine dinucleotide (NAD^+^) from nicotinamide mononucleotide (NMN; Araki et al., 2004). This protective role of Wld^*s*^ is conserved evolutionarily across diverse species (MacDonald et al., 2006; Martin et al., 2010).

Building on the exciting discovery that the Wld^s^ mutation slows the process of axon degeneration, multiple studies have addressed the roles of NMNAT enzymes in axonal survival. There are three mammalian NMNATs. Loss of function mutations in NMNAT2, but not the other NMNATs triggers Wallerian-like degeneration in undamaged axons (Gilley and Coleman, 2010; Gilley et al., 2013). As NMNAT2 is selectively expressed in axons and has a very short half-life, NMNAT activity in axons requires continued anterograde transport of this enzyme from the cell soma (Figure 1). Slowing the turnover of NMNAT2 can significantly delay injury-induced WD (Xiong et al., 2012; Babetto et al., 2013; Milde et al., 2013a,b). These studies on NMNAT2 highlight the essential role of axonal transport and proteasomal degradation in maintaining homeostatic protein content in axons. Disruption of either pathway can reduce any levels of critical axonal protein levels below a necessary threshold and so induce degeneration cascade. Therefore, chemotherapy-induced changes in axonal transport, translation and proteasomal degradation represent important avenues of investigation in future studies of CIPN.

Studies of axotomy have also identified SARM1, a scaffolding molecule, as a critical regulator of axonal degeneration. Loss of function of SARM1 significantly delays axon degeneration after injury, indicating that SARM1 functions as a pro-degeneration factor in WD (Osterloh et al., 2012; Gerdts et al., 2013). Depletion of SARM1 prevents the axon degeneration phenotype seen with NMNAT2 loss of function, indicating that these two components act in the same signaling cascade, with SARM1 downstream of NMNAT2 (Gilley et al., 2015). To understand the functions of SARM1 and NMNAT2, several investigations have analyzed the importance of NAD^+^ vs. NMN and ATP, the product and the substrates of NMNAT2 enzymatic activity, respectively. It is not yet clear whether loss of NMNAT2 triggers axon degeneration as a result of NAD^+^ deficiency, or accumulation of NMN, or other outcomes. Whether SARM1 alters NAD^+^ or NMN to induce axon degeneration is an area of active investigation (Di Stefano et al., 2015, 2017; Gerdts et al., 2015; Sasaki et al., 2016; Essuman et al., 2017). One important consequence of SARM1 activation or of NMNAT2 loss is activation of DLK/MAPK signaling, which ultimately triggers calpain-mediated axon degeneration (Yang et al., 2013, 2015; Figure 1).

Calpain and many additional components implicated in axonal responses to injury are linked to CIPN. Initial evidence of these similarities came from studies demonstrating that Wld^*s*^ mutant neurons display resistance to vincristine-induced neuropathy (Wang et al., 2001). More recently, SARM1 knockout mice were shown to be resistant to vincristine in an *in vivo* model (Geisler et al., 2016). Essuman et al. demonstrated that SARM1 functions as a NAD^+^-depleting enzyme and that the intrinsic NADase activity of SARM1 is required for vincristine-induced axon degeneration (Essuman et al., 2017). Additional evidence that the SARM1 pathway plays a critical role in CIPN includes data that expression of axonal NMNAT1 and constitutive expression of the NMN deaminase that consumes NMN, both protect against vincristine-induced peripheral neuropathy (Sasaki et al., 2009; Di Stefano et al., 2017). Therefore, inhibition of SARM1 enzymatic activity might be a useful therapeutic strategy in CIPN.

### Human variants

In addition to these models of development and axotomy, a final approach for identifying molecular cascades implicated in CIPN is to discover human variations that alter susceptibility to CIPN. The incidence of CIPN varies significantly from person to person. Increased age, diabetes, and previous neuropathy are known risk factors for CIPN. However, it is likely that genetic variations are also important for susceptibility. Several genome wide association studies (GWAS) have identified gene variants associated with increased vulnerability to CIPN. Single nucleotide polymorphisms of FGD4 and EPHA5 were significantly associated with susceptibility to paclitaxel-induced peripheral neuropathy in both European and African ancestral groups (Baldwin et al., 2012). FGD4 (FYVE, RhoGEF, and PH Domain Containing 4) is a Rho GTP/GDP exchange protein regulating actin cytoskeleton dynamics (Obaishi et al., 1998). Mutations of FGD4 in humans lead to autosomal recessive demyelinating Charcot-Marie-Tooth neuropathy (type 4H; De Sandre-Giovannoli et al., 2005; Delague et al., 2007; Stendel et al., 2007). The EPHA family is a group of tyrosine kinase receptors that bind to the Ephrin ligands. Eph/Ephrin signaling has been implicated in regulating axon outgrowth and guidance during development (Dickson, 2002; Egea and Klein, 2007). A recent study performed next generation sequencing on patients who developed significant neuropathy after paclitaxel, and identified variations of three related receptors, EPHA5/6/8, that correlated with susceptibility to paclitaxel-induced neuropathy (Apellaniz-Ruiz et al., 2017). Other CIPN associated genes identified by GWAS studies also play critical roles in neurite outgrowth and nerve development including FZD3 (Baldwin et al., 2012), TUBB2A (Leandro-Garcia et al., 2012), VAC14 (Hertz et al., 2016). These candidates may affect CIPN by directly affecting the cytoskeleton organization (TUBB2A, FGD4), by regulating intracellular trafficking (VAC14) or may function as signaling molecules regulating axon growth (EPHA, FZD3). Future verifications are required to confirm the involvement of these genes in CIPN pathology, and how these factors might contribute to the disorder.

## Concluding remarks

As summarized in this review, multiple chemotherapeutic agents that cause CIPN are directed against different molecular targets. Preclinical studies have generated several hypotheses to explain the pathogenesis of CIPN by these agents, including defective axon transport, mitotoxicity, and altered Ca^2+^ homeostasis. While each agent may initially affect the nervous system in a distinctive manner, the pathologies observed in CIPN converge to cause an axon degenerative process. Mechanistic studies of the phenotypically similar axon degeneration processes during developmental axon pruning or during injury-induced WD are likely to shed new light on the molecular basis of CIPN. Indeed, several signaling molecules on the SARM1, DLK/MAPK, and NMNAT pathways that protect axons from WD can also impact CIPN. Concurrently, human GWAS studies have identified pathways implicated in neurite outgrowth and/or axon maintenance that may be relevant for axon degeneration in CIPN. These findings suggest that axon degeneration is central to CIPN pathology. Enhanced understanding of the pathologic process of axon degeneration will be essential for developing an arsenal of effective therapies of CIPN.

## Author contributions

All authors listed have made a substantial, direct, and intellectual contribution to the work, and approved it for publication.

### Conflict of interest statement

The authors declare that the research was conducted in the absence of any commercial or financial relationships that could be construed as a potential conflict of interest.
